# Trends in Fruit Quality Improvement From 15 Years of Selection in the Apple Breeding Program of Washington State University

**DOI:** 10.3389/fpls.2021.714325

**Published:** 2021-10-18

**Authors:** Soon Li Teh, Sarah Kostick, Lisa Brutcher, Bonnie Schonberg, Bruce Barritt, Kate Evans

**Affiliations:** Tree Fruit Research and Extension Center, Washington State University, Wenatchee, WA, United States

**Keywords:** acidity, crispness, firmness, instrumental, juiciness, sensory, storability, sweetness

## Abstract

Washington State University's apple breeding program (WABP) was initiated in 1994 to select new apple cultivars with improved eating quality, appearance, and storability that are suitable for production in the main growing regions of the state. Fruit quality is phenotyped using various instrumental measures, such as penetrometers (texture), titrator (acidity), and refractometer (soluble solids concentration; SSC), as well as sensory assessment. The selection regime of WABP occurs in three sequential phases: phase one (P1)—single, unreplicated seedlings at one site, phase two (P2)—replicated selections at three geographically diverse sites, and phase three (P3)—highly replicated elite selections at one to two grower sites. Most of the data collection of WABP occurs in P2. Knowledge of trends/changes associated with advancing selections is essential for understanding the selection criteria and progress of WABP throughout the changing compositions of advancing and culling selections. For each post-harvest trait, P2 data from harvest years 2005 to 2019 were split across sites, and between selections and reference cultivars (e.g., Cripps Pink, Gala, and Honeycrisp). Means of instrumental crispness (Cn) and inner cortex firmness for the advancing selections increased gradually over this period and were significantly higher than those for cultivars. Means of outer cortex firmness measurements were stable for selections but significantly higher than those for cultivars. The average fruit acidity of selections increased marginally over this period and was higher than that of the cultivars. Meanwhile, the average fruit SSCs of selections and cultivars were statistically indistinguishable. These 15-year trends indicate that WABP has been selecting apples with improved eating quality and storability through increased crispness and inner cortex firmness, respectively.

## Introduction

The Washington State University apple breeding program (WABP) was initiated in 1994 to develop new, improved apple cultivars that are suitable for production in the main growing regions of the state and that are available to all commercial Washington apple growers. The primary breeding targets of WABP are improved eating quality, appearance, and storability (Evans, 2013). The Washington state leads the nation with ~70% of US apple (*Malus domestica*) production (Washington Apple Commission., 2021). In 2019–2020, none of the top five production volume apple cultivars (i.e., Gala, Red Delicious, Fuji, Granny Smith, and Honeycrisp) grown in the Washington state was originally bred for the state (Washington State Tree Fruit Association., 2021).

The selection regime of WABP occurs in three sequential phases after seedling production in the greenhouse. In phase one (P1), unreplicated seedling trees are planted at one research site. Fruit sampling begins in the sixth year when 10 fruits from trees with desirable appearance and eating quality are harvested at Cornell starch-iodine indices between three and five (Blanpied and Silsby, 1992) and stored in the regular atmosphere (RA) at 1–2°C for 2 months prior to assessment. Promising unreplicated seedling trees are tested more fully in years 7 and 8 with increased fruit samples for 1–2°C RA storage of two and four months prior to assessment. The best P1 seedlings are selected and propagated for planting in phase two (P2).

Five clones of each P2/advanced selection are planted in a randomized block design with reference cultivars (e.g., Cripps Pink, Gala, and Honeycrisp) at each of the three geographically diverse sites in Washington State. Up to 30 fruits are harvested weekly starting at Cornell starch-iodine index of three (Blanpied and Silsby, 1992), and continuing for up to 3 weeks. Similar to P1, fruit quality is evaluated at harvest, after 2 and 4 months of 1–2°C RA storage. In addition to fruit quality traits, various horticultural traits are phenotyped during P2, the principal data collection phase of WABP. Advanced selections that perform well in P2 are propagated for more extensive planting and evaluation in phase three (P3).

Fruit quality assessment is critical for determining the eating quality and storability of post-storage fruits in WABP. Fruit quality is a multi-faceted description that includes various traits, such as texture. Owing to its influence on consumer acceptance (Harker et al., 2003, 2006; Harker et al. 2008, 2008), fruit texture is a primary breeding target. Fruit quality evaluations in WABP consist of instrumental analyses and sensory assessments. The former includes fruit size, fruit weight, and a myriad of textural traits analyzed with a computerized penetrometer, as previously detailed by Evans et al. (2010) and Teh et al. (2020b). Sensory assessment is carried out by four long-term WABP team members using a list of predefined sensory attributes (e.g., acidity, crispness, hardness, juiciness, and sweetness) to assess a pool of five fruits (Evans et al., 2010, 2012a; Teh et al., 2020b).

Washington State University's apple breeding program has amassed data of various post-harvest traits over the past 15 years of routine evaluation and selection. Knowledge of trends and changes associated with advancing selections is pivotal for understanding selection criteria and the progress of WABP throughout the changing compositions of advancing and culling trees. Such investigation is typical in major annual crops, such as corn (Lauer et al., 2012), soybean (Voldeng et al., 1997; Morrison et al., 1999, 2000; Ustun et al., 2001; Wilcox, 2001; Jin et al., 2010; Rowntree et al., 2013), and winter wheat (Donmez et al., 2001), where yield indices were compared among cultivars of different years of release to characterize the improvement of new cultivars over the predecessors. However, such investigations on perennial fruit crops, such as apples, are complicated by their perenniality. At WABP, as with other apple breeding programs, trees/selections remain in an orchard for several years of evaluations. The continual process of culling undesirable selections and adding new selections results in a dynamically changing set of evaluation germplasm over time. In addition, the composite criteria of fruit quality (instead of yield indices in most annual crops) complicate any attempt to characterize trends and changes associated with advancing selections. To our knowledge, no previous work describing trends and selection progress in a perennial tree fruit breeding program is available.

The objective of this study was to characterize trends and changes of various post-harvest traits throughout the 15 years of selection by WABP. Instrumental and sensory assessment data of P2 selections collected through the routine operation of WABP from 2005 to 2019 were analyzed to describe year-to-year trends across multiple post-harvest traits.

## Materials and Methods

### Experimental Material

From 2005 to 2019, apples from 143 P2 selection accessions (crosses from 1994 to 2008) and 35 reference accessions were harvested from five geographically distinct WABP evaluation orchards in central Washington. All accessions within each site were managed similarly although there were slight differences in management styles between the sites, especially those of grower collaborators. Depending on the planting year and rootstock availability, accessions were propagated on several different M9 rootstock clones or on Geneva 41. Crop load was managed following the “Young Apple Thinning Gauge” guide (Miranda Sazo, 2014). The 35 reference accessions consisted of eight unique cultivars, namely Braeburn, Co-op 15, Cripps Pink, Fuji, Gala, Golden Delicious, Honeycrisp, and Scifresh planted in different years to coincide with selection plantings (Table 1). The combined data sets of 178 selections/accessions represent all fruits harvested as part of the WABP routine evaluation of fruit quality from 2005 to 2019.

**Table 1:** Reference cultivars represented in the phase two evaluation of the Washington State University apple breeding program for harvest years from 2005 to 2019.

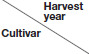	**2005**	**2006**	**2007**	**2008**	**2009**	**2010**	**2011**	**2012**	**2013**	**2014**	**2015**	**2016**	**2017**	**2018**	**2019**
Braeburn	1	1	1	1	1	1	1	1	1	–	–	–	–	–	–
Co-op 15	1	1	–	–	–	–	–	–	–	–	–	–	–	–	–
Cripps Pink	1	–	2	2	2	3	2	5	6	5	5	3	3	4	3
Fuji	1	1	2	2	2	3	1	5	6	5	5	3	4	4	3
Gala	1	2	3	3	3	4	5	6	7	5	5	2	2	2	3
Golden Delicious	–	1	1	1	1	3	2	4	5	6	6	3	3	3	3
Honeycrisp	–	–	–	–	–	1	1	3	4	5	5	3	3	3	4
Jazz	–	–	–	1	1	1	–	–	–	–	–	–	–	–	–

*Numeric values denote the number of accessions for each cultivar of different planting years (1994–2008). For example, seven Gala accessions of different planting years were evaluated in 2013. Fruits were sampled from each accession up to three harvest dates at up to three locations (i.e., North, Central, and South) each year*.

The harvest regime of WABP was described in detail in two recent studies (Teh et al., 2020a,b) and is summarized here for one selection/accession. Since the optimum harvest parameters of a selection are unknown, fruits were harvested when starch levels approximated the indices of three to five, based on the Cornell starch-iodine index test (Blanpied and Silsby, 1992), up to three harvests over 3 weeks. At each harvest, up to 30 fruits were harvested depending on the fruit availability. The first subset of 10 fruits was evaluated within a week of harvest. The second and third subsets of 10 fruits each were stored in the regular atmosphere (RA) at 1–2°C for 2 and 4 months, respectively. From the 2012 season onwards, fruits from the second and third subsets were held at room temperature of ~25°C for 1 week prior to fruit quality evaluations. Note that only the second subset of fruit (henceforth, storage fruit) is relevant to the focus of this study.

### Instrumental Measurements

From each 10-fruit storage sample, one subset of five apples was subjected to instrumental measurements, while the other subset of five was subjected to sensory assessment. Instrumental measures of fruit quality consisted of various textural traits, soluble solids content (SSC), and titratable acidity (TA). In one pool of five fruits, an intermediate (i.e., not sun or shade) side of each fruit was peeled and subjected to instrumental texture analysis using a penetrometer. From 2005 to 2008, a TA-XT2 Texture Analyzer (Texture Technologies, Scarsdale, NY, USA) with a standard mechanized Magness-Taylor 11-mm probe was used to measure the firmness of the outer cortex fruit. From 2009 to 2013, Mohr™ Digi-Test Model 1 (MDT-1; Mohr and Associates Inc., Richland, WA, USA), a computerized penetrometer with an 11-mm diameter probe was used to measure various textural traits (Evans et al., 2010; Teh et al., 2020b). From 2014 to 2019, Mohr™ Digi-Test Model 2 (MDT-2) was used in replacement of MDT-1. No statistical difference was detected in fruit textural outputs between two sequential models of the computerized penetrometers during the transition of WABP from MDT-1 to MDT-2 (Teh et al., 2020b).

Mathematical interpretation of texture analysis outputs is based on a two-region anatomy model of peeled apple fruit. A fixed 8-mm depth from the outermost region of the fruit cortex is defined as region 1 (R1), which is the region measured with industry standard Magness-Taylor-type penetrometers (Magness and Taylor, 1925). Beneath R1, region 2 (R2) covers the bulk of edible cortex material between R1 and the core tissue (Mohr and Mohr, 2007). Penetrometer textural outputs relevant to this work are maximum hardness in R1 (M1), maximum hardness in R2 (M2), and instrumental crispness (Cn), a unitless calculation derived from force data in the mid-region of the fruit as the penetrometer plunger moves between R1 and R2 with constant velocity, which estimates the energy released during fruit tearing (Mohr and Mohr, 2007).

Following texture measurements, the punctured fruits were sliced horizontally and inspected for internal disorders. Sliced fruits were cut into chunks, and up to 12 chunks from each of the five apples were pooled and juiced. Several drops of juice were dispensed on a refractometer (RX-5000α-Bev, ATAGO USA, Inc., Bellevue, WA, USA) to measure SSC. TA was measured with an automated titrator, Metrohm^®^ 815 Robotic USB Sample Processor XL (Metrohm, Riverview, FL, USA) based on an aliquot of 5 mL apple juice.

### Sensory Assessment

For each sample, the second subset of five fruits was subjected to sensory assessment, which was previously described by Evans et al. (2010) and Teh et al. (2020b). Sensory attributes (e.g., acidity, crispness, hardness, juiciness, and sweetness) were scored on a 5-point scale, with “1” and “5” being the weakest and strongest perceptions, respectively, by four core and long-term WABP team members. Sensory texture attributes (e.g., crispness and hardness) were rated following the descriptions by Harker et al. (2002). “Hardness” is characterized as a force exerted to completely bite through a sample between molars, while “crispness” as an acoustic sensation detected by the ear during food biting or tearing (Harker et al., 2002; Evans et al., 2010; Teh et al., 2020b). While scored on a 5-point scale, “hardness” and “crispness” were rated with reference to ‘Gala’ having a standard score of 3. Overall eating quality was scored on a 9-point scale with ‘Gala’ having a standard score of 5. All sensory ratings were determined on a pooled sample of five fruits. Scores for each sensory attribute from the four team members were averaged prior to statistical analysis.

### Statistical Analysis

The 15-year data set of 178 P2 selections/accessions (up to five replicate trees at each site) contains a total of 1,738 samples. Each sample represents fruits from a P2 selection/accession harvested at a particular WABP orchard on a particular harvest date of a year. The corresponding data points are averaged instrumental trait values from each of the five fruits or averaged sensory scores for pooled fruits from four team members.

All calculations, analyses, and plots were carried out in R version 4.0.3 (R Core Team., 2015) using RStudio version 1.4.1103 (RStudio Team., 2020). The data set was split between selections (i.e., 143 selections; 1,166 samples) and references (i.e., 35 references; 572 samples). Due to expected environmental variance, each data set was further split into subsets based on the North, Central, and South orchard sites. Annual averages and standard errors for each instrumental trait as well as sensory attributes were calculated to describe the trends from 2005 to 2019. Trend analyses and figures for trends were performed using *ggplot2* package version 3.3.3 (Wickham, 2016). All figures were compiled and rendered in Adobe Illustrator CS2 (Adobe, San Jose, CA).

Correlation analyses were performed on the entire data set for traits with related instrumental and sensory traits, such as SSC-sweetness, TA-acidity, M1-hardness, M2-hardness, and Cn-crispness. Spearman's rank correlation was used as a non-parametric measure of coefficients. Significance levels for Spearman's correlation were set at *P* ≤ 0.001. Spearman's coefficients were computed with the *rcorr* function from the *Hmisc* package version 4.4-2 (Harrell, 2017).

Principal component analysis (PCA) was conducted on the mean values of instrumental and sensory traits for 978 samples of 126 P2 selections from 2009 to 2019. Samples were restricted to this period because Cn and M2 were unavailable prior to 2009. Eating quality is a covariate trait included to determine the influence and correlation of other traits. PCA was carried out using FactoMineRpackage version 4.0.4 (Lê et al., 2008) and factoextra package version 4.0.4 (Kassambara and Mundt, 2017).

## Results

### Instrumental Analyses

**Figure 1:**
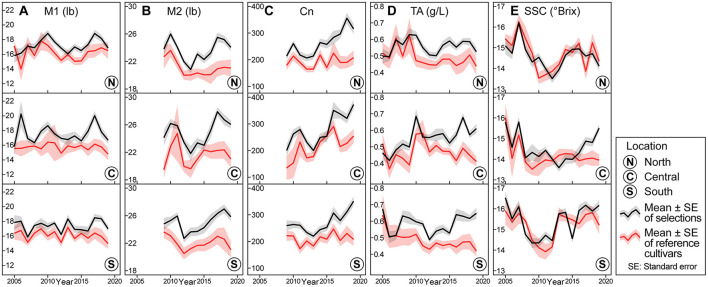
Trends of instrumental fruit quality traits for Washington State University apple breeding program for phase two selections and reference cultivars from 2005 to 2019. Means (as lines) and standard errors (as shades) of selections (in black), and cultivars (in red) for **(A)** M1—maximum hardness at region one, **(B)** M2—maximum hardness at region two, **(C)** Cn—instrumental crispness, **(D)** TA—titratable acidity, and **(E)** SSC—soluble solids content.

In general, across the three sites and all traits, standard errors for the means of phase two (P2) selections were significantly smaller than those of the cultivars. Maximum hardness in region 1 (M1) is the industry standard measure for outer cortex fruit firmness. Throughout the 15-year period, M1 means of phase two (P2) selections were generally higher than those of cultivars (fluctuating around equilibrium values of ~17 lbs and 16 lbs, respectively (Figure 1A). In comparison with maximum hardness in R2 (M2) or the inner cortex fruit firmness measure, M1 trends are more stable across sites throughout this period. The collection of M2 data began in 2009; prior data were unavailable. M2 trends of P2 selections are significantly higher than those of references. While cultivar M2 means generally stagnated from 2015 to 2019, those of P2 selections tended to increase during this period across the three sites (Figure 1B).

Instrumental crispness (Cn) is the output that describes crispness. Similar to M2, the collection of Cn data began in 2009; thus, prior data were unavailable. In all sites, Cn mean values of P2 selections were significantly higher than those of the cultivars. Specifically, Cn means of P2 selections increased starting from 2012 and peaked at ~350, while those of the cultivars fluctuated around 200 (Figure 1C).

From 2005 to 2008, titratable acidity (TA) means of P2 selections and references were similar. Thereafter, TA means of P2 selections were significantly higher than those of references. Average TA values of P2 selections in the Central and South sites showed a general increase over time, while those in the North site appeared to level off at ~0.55 g/L (Figure 1D).

Throughout the 15-year period, soluble solids concentration (SSC) means and standard errors of both the selections and references showed significant overlaps, indicating that SSC averages of these two groups were similar across the three sites. In general, SSC means of both the groups decreased from 2005 to 2012 but increased thereafter (Figure 1E).

### Sensory Analyses

**Figure 2:**
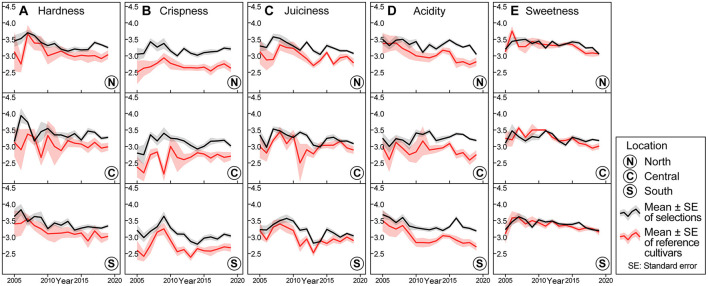
Trends of sensory assessed fruit quality traits for Washington State University apple breeding program for phase two selections and reference cultivars from 2005 to 2019. Means (as lines) and standard errors (as shades) of selections (in black), and cultivars (in red) for **(A)** hardness **(B)** crispness, **(C)** juiciness, **(D)** acidity, and **(E)** sweetness.

Similar to the overview observation made in instrumental analyses, standard errors for the means of all sensory traits of P2 selections were significantly smaller than those of cultivars across the three sites. Means of sensory hardness for selections and cultivars were stable. Average hardness values of selections were marginally higher than those of references (Figure 2A).

For sensory crispness, means of selections were significantly higher than those of references throughout all sites (Figure 2B). In contrast to the general increase exhibited in instrumental crispness (Cn) means, the average sensory crispness values of selections stagnated between 2.9 and 3.2 (Figures 1C, 2B). Trends for sensory juiciness more closely resemble trends for sensory hardness, where the means of the selections were marginally higher than the means of references (Figures 2B,C).

As for sensory acidity, means and standard errors of selections and references overlapped through 2009. Thereafter, sensory acidity means of selections were consistently higher than those of references across the three sites. Average sensory acidity values of selections generally stagnated at a score of 3.4 (Figure 2D). The sensory acidity trend was comparable with the TA trend (Figures 1D, 2D).

Throughout most of the 15-year period, means of sensory sweetness for selections and references showed significant overlaps across the three sites, a trend consistent with the SSC line plots. However, the sensory sweetness means for selections and references stagnated between 3.2 and 3.4, while instrumental SSC means showed significant fluctuations throughout (Figures 1E, 2E).

### Correlation and Multivariate Analyses

In the correlation analyses of all samples (*n* = 1,738), TA, M1, and M2 were correlated with their sensory counterparts (acidity and hardness, respectively) at moderately high coefficients (*r* = 0.71, 0.68, and 0.61, respectively) (Table 2). Cn was moderately correlated with sensory crispness (*r* = 0.46) and sensory juiciness (*r* = 0.36).

**Table 2:** Spearman's rank correlation of instrumental traits and corresponding sensory traits for **(A)** all samples, **(B)** phase two selections only, and **(C)** reference cultivars only from Washington State University apple breeding program.

**A**	**All (1,738 samples)**		**Instrumental traits**				
			**SSC**	**TA**	**M1**	**M2**	**Cn**
	Sensory traits	Sweetness	0.28				
		Acidity		0.71			
		Hardness			0.68	0.51	
		Crispness					0.46
		Juiciness					0.36
**B**	**Selections (1,166 samples)**		**Instrumental traits**				
			**SSC**	**TA**	**M1**	**M2**	**Cn**
	Sensory traits	Sweetness	0.22				
		Acidity		0.57			
		Hardness			0.62	0.54	
		Crispness					0.29
		Juiciness					0.15
**C**	**References (572 samples)**		**Instrumental traits**				
			**SSC**	**TA**	**M1**	**M2**	**Cn**
	Sensory traits	Sweetness	0.37				
		Acidity		0.74			
		Hardness			0.77	0.71	
		Crispness					0.50
		Juiciness					0.56

*All coefficients are at significance levels of P < 0.001*.

When the overall data set was split between selections and cultivars, the correlation coefficients of the latter were higher than those of the former. For instance, coefficients of TA-acidity, M1-hardness, and M2-hardness of references were 0.74, 0.77, and 0.71 respectively, while those of the selections were 0.57, 0.62, and 0.54, respectively (Table 2).

**Figure 3:**
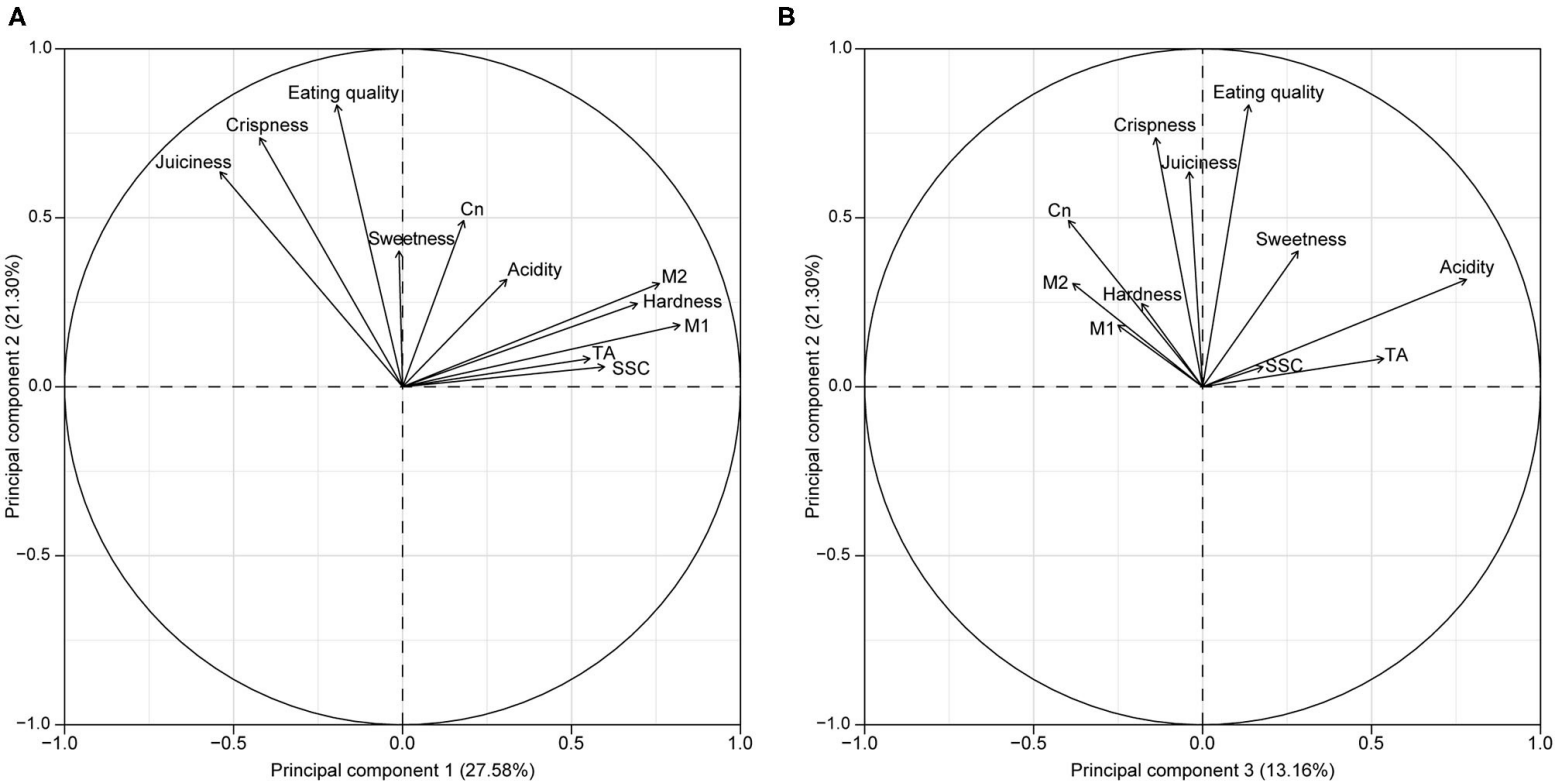
Variable factor maps of principal component analysis (PCA) for instrumental and sensory fruit quality traits of Washington State University apple breeding program for phase two selections from 2009 to 2019. Variable factor maps for **(A)** PC1 and PC2 and **(B)** PC2 and PC3.

In the principal component analysis (PCA), variable factor map between PC1 and PC2, sweetness, crispness, Cn, juiciness, and acidity are the top five variables/traits that are most positively correlated with eating quality. The remaining five variables (i.e., hardness, M1, M2, SSC, and TA) are tightly clustered, indicating high correlations with each other (Figure 3A). Similarly, in the PCA map between PC2 and PC3, juiciness, crispness, and sweetness are more strongly clustered with eating quality. In quadrant one, the vectors for sweetness and acidity are in close proximity to their respective instrumental vectors. In quadrant two, the vectors for hardness, Cn, M1, and M2 converge tightly, indicating high correlations among these variables (Figure 3B).

## Discussion

This work summarizes selection progress and trends in fruit quality from 2005 to 2019 using data routinely collected during fruit evaluations at the Washington State University apple breeding program (WABP). This study drew inspiration from previous reports of genetic gains in annual crops (Voldeng et al., 1997; Morrison et al., 1999, 2000; Donmez et al., 2001; Ustun et al., 2001; Wilcox, 2001; Jin et al., 2010; Lauer et al., 2012; Rowntree et al., 2013), but differs in the types of crops (annual vs. perennial) and traits of interest. In most annual crops, selection progress is largely gauged by yield and yield indices as the principal breeding targets. Yield in apple is routinely manipulated by rootstock, planting density, and bloom or fruitlet thinning (Musacchi and Greene, 2017). Fruit quality is typically the primary breeding target for apples; however, the multi-facets of fruit quality and the interplay between traits complicate the characterization of selection progress and genetic gains. To our knowledge, this report is the first attempt in characterizing the selection trends in a perennial fruit crop breeding program.

Due to the complexity of fruit quality, most instrumental traits [e.g., instrumental crispness (Cn), soluble solids concentration (SSC), and titratable acidity (TA)] are supplemented with sensory assessment (e.g., crispness, sweetness, and acidity, respectively) to provide a human element/factor during fruit evaluations at WABP. In addition, the fruit quality of breeding selections is routinely compared with cultivars, which provide baseline information regarding fruit texture, appearance, and storability. Since these references are distinctively different from each other, their overall trait variances (presented as standard errors) were larger than those of P2 selections. For instance, ‘Cripps Pink’ is a firm and high-acid apple, while a ‘Golden Delicious’ apple is lower in firmness and acidity.

### Higher Inner Cortex Firmness Among Selections

Over the 15-year period, our results indicated that WABP selected for apples with marginally higher outer cortex firmness (i.e., M1) but that retained significantly higher inner cortex flesh firmness (i.e., M2) after the 2-month storage regime. Physiological deterioration (e.g., softening) during storage progresses from the core outwards (Rudell et al., 2000); therefore, a higher M2 value is indicative of longer storability. Such improvement was evidenced in our results based on the two patterns exhibited among WABP P2 selections since 2012: (1) M2 means increased; (2) M2 means of selections were significantly higher than those of the references. The sensory counterpart for M1 and M2 is sensory hardness/firmness. P2 trends for sensory hardness more closely resemble M1 trends than M2. This observation was empirically supported by the correlation results, where sensory hardness is more highly correlated with M1 (selections *r* = 0.62; references *r* = 0.77) than M2 (selections *r* = 0.54; references *r* = 0.71). During the sensory assessment, the perception of firmness may be more influenced by the outer cortex fruit firmness than the inner cortex flesh firmness.

### Higher Crispness Among Selections

In addition to the threshold of firmness, crispness is the most desired textural characteristic valued by both the consumers (Harker et al. 2008, 2008) and producers (Yue et al., 2013), and is, therefore, a major target for WABP. Here, our results indicated that WABP selected for apples with higher crispness. In the principal component analysis (PCA) variable factor map, eating quality is more positively correlated with Cn and sensory crispness. The increase in crispness among P2 selections, particularly since 2012, might be attributed to the increased use of cultivar, Honeycrisp (known for its “explosive” crispness; Luby and Bedford, 1992) as a breeding parent (data not shown). While these trends were observed in the instrumental results, such trends were not immediately apparent in the sensory counterpart. The discrepancy between increasing instrumental Cn means and stable sensory crispness means can be explained by several reasons. First, instrumental Cn is a quantitative measure, while sensory crispness is rated on a 5-point scale. Second, as the proportion of crisp apples increased over the years, the perception of the crispness was likely not as pronounced as in earlier years. Third, the perception of crispness might be affected by the firmness of flesh. It can be easier to perceive crispness in a softer apple than in a firmer apple, thus impacting the ratings. Fourth, the perception of crispness might be impacted by the thickness of apple skins. In a recent study by Bejaei et al. (2021), crispness and skin toughness/thickness were reported to be negatively correlated (*r* = −0.27), indicating that apples with thinner skin had higher perceived crispness levels, all others constant. At WABP, fruit textural measurements were acquired from a peeled fruit (as required by the penetrometer), while the sensory assessment was performed by biting through an unpeeled fruit. Though experienced team members made every effort to perceptively account for crispness, flesh firmness, and skin thickness, human subjectivity in routine assessments of up to 100 samples might be a factor. Despite these differences, results from sensory crispness corroborated instrumental measurements that the crispness of P2 selections was significantly higher than that of the cultivars.

### Juiciness Trends of Selections Indeterminate

Sensory juiciness is a complex trait due to the physicochemical properties of fruit (e.g., the ratio of air spaces to total tissue volume, cell wall thickness, cell dimensions, as well as water distribution in intracellular, intercellular, and cell walls) that can impact the perception of juiciness (Joardder et al., 2015; Iwanami et al., 2017). In the PCA variable factor map, juiciness and crispness were highly correlated. Similarly, previous studies reported sensory juiciness to be moderately (Evans et al., 2010; Zdunek et al., 2010; Teh et al., 2020b) to highly correlated (Cliff and Bejaei, 2018) with sensory crispness. In correlating sensory juiciness with instrumental Cn, correlation coefficients ranged from low to moderate, with *r* values of 0.36 (Evans et al., 2010), 0.72 (Cliff and Bejaei, 2018), and 0.33 (Teh et al., 2020b). The range in coefficients could be partially explained by the diversity of germplasm used, wherein the lower correlation coefficients are expected in a more diverse set of germplasm. Spearman's coefficients between sensory juiciness and instrumental Cn were moderate (*r* = 0.56) among cultivars, but low (*r* = 0.15) among the P2 selections. The former consisted of 572 samples from only eight unique cultivars, while the latter consisted of 1,166 samples from 143 unique P2 selections.

### Marginally Increased Acidity Among Selections

Both trends and correlation analyses between the sensory acidity and TA showed that sensory and instrumental acidity were moderately correlated (0.57 ≤ *r* ≤ 0.74), indicating that the perception of acidity was discriminating and reliable. The higher coefficient (*r* = 0.74) among cultivars compared to that (*r* = 0.57) of P2 selections was expected and might be explained by the more distinctive differences in acidity among cultivars; “Cripps Pink” is a high-acid apple, while “Gala” has a lower acidity profile (Evans et al., 2012b).

The total organic acid content in apples plays a pivotal role in the overall eating experience and post-harvest storability (Musacchi and Serra, 2018). Malic acid, the predominant organic acid in apple, is a major substrate for enzymatic respiration during storage, resulting in the loss of malic acid and higher permeability of the inner cortex (Hulme et al., 1963). Over the 15-year period, WABP selected for apples with acidity levels higher than the average of cultivars after storage. The separation between P2 selections and cultivars became more apparent from 2012 across the three locations, aligning with the addition of 1 week at 25°C after refrigerated storage. Retention of malic acid levels and inner cortex firmness were typically higher in selections than in the cultivars. While the TA levels of recent selections were not as high as of ‘Cripps Pink,’ they were significantly higher than those of ‘Fuji,’ ‘Gala,’ and ‘Golden Delicious’ (data not shown).

### SSCs of Selections Comparable to That of the Cultivars

In addition to acidity, sweetness is an important flavor trait, and balance between acidity and sweetness is pivotal in the consumer perception of the desirable eating quality in apples (Iwanami, 2011). Over the 15-year period, SSC trends fluctuated, but average SSCs of selections were comparable to those of cultivars. Similarly, trends of sensory sweetness were relatively indistinguishable between selections and cultivars, albeit with less fluctuation. These sensory trends are weakly correlated (0.22 ≤ *r* ≤ 0.37) with the instrumental SSC trends, consistent with the values (0.22 ≤ *r* ≤ 0.35) reported by Kouassi et al. (2009), but significantly lower than *r* = 0.64 reported by Aprea et al. (2017). The weak correlation could be due to several reasons. First, sweetness perception might be masked by acidity levels, where a high-acid apple is perceived lower in sweetness than a low-acid apple, all others constant. This is consistent with a previous report by Aprea et al. (2017), noting the negative effect of malic acid on perceived sweetness. Second, SSC is an indirect estimate of soluble sugars that are commonly used in many breeding programs for phenotype sweetness (Evans, 2013), but the primary sugars in apples consist of fructose, glucose, and sucrose, as well as sorbitol, sugar alcohol (Fuleki et al., 1994). Each individual sugar/sugar alcohol has varying effects on the perceived sweetness, and the combination of sugars on sweetness perception remains largely unknown. Aprea et al. (2017) reported that sorbitol explained 44% of sweetness perception, whereas total sugars (fructose, glucose, sucrose, and xylose) explained only 17%. Third, large differences in SSC trends across the three locations correspond to SSC being a highly quantitative trait that is controlled by multiple small-effect loci, resulting in a strong genetic × environment interaction. Guan et al. (2015) and Ma et al. (2016) reported multiple genetic loci associated with individual sugars on 11 of the 17 haploid chromosomes in the apple genome. Taken together with acidity, this indicates that WABP has been selecting apples with SSC/sweetness levels that are comparable to those of elite cultivars, but with marginally higher levels of acidity.

### Limitations

A limitation to this investigation stems from the perenniality of the apple. Since apple selections remain in a research orchard for multiple years of evaluation, and crop load and quality can vary with the age of the tree (Stefanelli et al., 2018), comparing a selection planted in 1 year is empirically dissimilar with another selection planted in a different year. Even when multiple selections were planted in the same year, some might be culled earlier than others. Due to different planting years and rootstock availability, selections were propagated on different rootstocks, exposed to various weather conditions, and subjected to slightly different horticultural management practices. In addition, tools and instruments for evaluation may change, potentially rendering results/outputs more difficult to compare. Fortunately, the technological transition from computerized penetrometer Mohr™ Digi-Test Model 1 (MDT-1) to Mohr™ Digi-Test Model 2 (MDT-2) was determined to be statistically insignificant (Teh et al., 2020b); however, no data exist to make a similar comparison between the TA-XT2 Texture Analyzer and the MDT-1. Slight changes in the protocol are inevitable over this time span. In 2012, WABP introduced a one-week storage at room temperature to all samples following cold storage to better evaluate the potential for selections to maintain quality for the consumer. The results of this protocol adjustment are evidenced by the substantial declines in M1 and M2 values post 2012 (Figures 1A,B). These inherent challenges with a perennial fruit crop are in stark contrast to a genetic gains study of an annual crop, where cultivars spanning several years or even decades of release can be planted simultaneously and harvested in the same year, as shown in the studies by Voldeng et al. (1997), Morrison et al. (1999), Morrison et al. (2000), Donmez et al. (2001), Ustun et al. (2001), Wilcox (2001), Jin et al. (2010), Lauer et al. (2012), and Rowntree et al. (2013).

Another potential limitation of this study was the uneven representation of reference cultivars (Table 1). Although eight cultivars were represented throughout, only two cultivars, namely Fuji and Gala were available, harvested, and evaluated during each of the 15 years. Cultivars like Braeburn and Co-op 15 were harvested in the earlier years but were subsequently replaced by cultivars that were more representative of the current Washington State crop, such as Honeycrisp, which was included in the cultivars set from 2010. Due to the differences in taste and texture among the cultivars, cultivar trends should be interpreted with additional consideration. For instance, post-2010 marginal increases in Cn means of cultivars were likely attributed to the inclusion of Honeycrisp, a crisp apple in the cultivars set. However, such interpretation is not as straightforward when observing the cultivar trend of sensory crispness, which showed stable means post-2010.

### Impact of Qualitative and Quantitative Traits on Fruit Quality Improvement

Based on the trends observed in this study, it is most apparent that SSC trends exhibited the largest variability across locations, indicative of a highly quantitative trait. Since SSC (i.e., sugar estimate) is controlled by multiple small-effect loci (Guan et al., 2015; Ma et al., 2016), directional selection for increased SSC is likely gradual and incremental. Instrumental acidity trends showed less variability across locations. Two genetic loci (*Ma* and *Ma3*), associated with a malic acid content, are known to jointly explain up to 66 ± 5% variance (Maliepaard et al., 1998; Kenis et al., 2008; Ma et al., 2016; Verma et al., 2019). Thus, the higher acidity levels exhibited by the fruit of selections are likely through introgression (i.e., positive selection) of high acidity alleles from parents, such as “Cripps Pink” (*Ma3*/*ma3, Ma*/*ma*; Verma et al., 2019).

Fruit texture is a complex trait characterized both mechanically and acoustically. While loci associated with texture have been reported on virtually all 17 apple chromosomes (Teh et al., 2021), a handful of major genes, namely *Md-PG1* (firmness and storability; Longhi et al., 2013), *Md-ACS1*, and *Md-ACO1* (ethylene production and softening; Zhu and Barritt, 2008; Baumgartner et al., 2016) are highly associated with texture and storability. The use of breeding parents with favorable texture alleles would increase the likelihood of desirable fruit texture of seedlings/selections. Although crossing to produce the selections (1994 to 2008) described in this study pre-dated the availability of DNA tests, subsequent analysis revealed that many of the breeding parents used included favorable texture alleles (data not shown). Further detailed analysis of the allelic representation of the WABP parent set is ongoing and is beyond the scope of this study.

### Conclusions

This study describes the progress on WABP and trends in fruit quality traits made from 2005 to 2019 that were enabled through strategic structuring of phenotypic data and statistical framework. Splitting data sets based on three geographically distinct regions (i.e., North, Central, and South) minimizes any extraneous variability associated with locations. Partitioning P2 selections from cultivars provide a frame of reference for tracking the progress of evaluated selections compared to commercial cultivars. Finally, complementation of instrumental outputs with sensory assessment, albeit imperfect, provides a relatable, “human” overall eating experience that ultimately guides breeding decisions. In summary, this work describes 15-year trends of fruit quality traits that were measured instrumentally and sensorially at WABP. The selection progress indicates that WABP has been selecting apples with increased storability (evidenced from increased internal flesh firmness), as well as improved eating quality from increased crispness, marginally higher acidity, and SSC/sweetness comparable to existing commercial cultivars.

## Data Availability Statement

The raw data supporting the conclusions of this article will be made available by the authors, upon request.

## Author Contributions

SLT, SK, and KE designed the project. KE and BB managed the breeding program. KE, BB, LB, and BS collected data. SLT conducted statistical analyses and wrote the manuscript. All authors contributed to the final draft and approved the submitted version.

## Funding

This work was supported by the USDA National Institute of Food and Agriculture Hatch project 1014919, Crop Improvement and Sustainable Production Systems (WSU reference 00011).

## Conflict of Interest

The authors declare that the research was conducted in the absence of any commercial or financial relationships that could be construed as a potential conflict of interest.

## Publisher's Note

All claims expressed in this article are solely those of the authors and do not necessarily represent those of their affiliated organizations, or those of the publisher, the editors and the reviewers. Any product that may be evaluated in this article, or claim that may be made by its manufacturer, is not guaranteed or endorsed by the publisher.
